# Transverse Split Laminoplasty: A Novel Anatomy Preserving Technique

**DOI:** 10.7759/cureus.36519

**Published:** 2023-03-22

**Authors:** Daphne Li, David H Hong

**Affiliations:** 1 Pediatric Neurosurgery, Advocate Lutheran General Hospital, Park Ridge, USA; 2 Neurosurgery, Loyola University Medical Center, Maywood, USA; 3 Pediatric Neurosurgery, Lehigh Valley Health Network, Allentown, USA

**Keywords:** surgery spine, ultrasonic bone scalpel, pediatric spine surgery, posterior tension band, laminoplasty

## Abstract

Laminoplasty is a technique often used in spine surgery as a way of restoring a patient’s normal anatomy and posterior tension band (PTB). In pediatric patients, this is an important consideration given their intrinsic relative ligamentous laxity and significant potential for future growth. Conventional laminoplasty re-approximates the bone removed at each vertebral segment, relying on the lamina to heal through osteogenesis and the soft tissue of the PTB to heal from a sharp division. Ligamentous healing of the PTB constitutes the formation of a scar that is biologically and biomechanically inferior to the tissue it has replaced. Herein, the authors present two pediatric cases in which the novel technique of transverse split laminoplasty was used to approach intradural pathology and subsequently reconstruct the spine, while maximizing preservation of the PTB, with excellent post-surgical outcomes.

## Introduction

Laminoplasty is a technique commonly used in spine surgery as a method of restoring a patient’s normal anatomy and posterior tension band (PTB). Conventional laminoplasty re-approximates the bone removed at each vertebral segment, relying on the lamina to heal through osteogenesis, while the soft tissue of the PTB must heal from the sharp division at either or both rostral and caudal aspects of the laminoplasty segment. Unfortunately, ligamentous healing results in the formation of a scar that is biologically and biomechanically inferior to the tissue it has replaced [1]. In pediatric patients, this is an important consideration, given their intrinsic relative ligamentous laxity and significant potential for future growth. We present two cases of pediatric patients who underwent multilevel laminoplasties for surgical management of intradural spinal pathology through a novel transverse split laminoplasty technique (TSL) with excellent healing and post-surgical outcomes.

## Case presentation

CASE #1

Clinical Presentation

**Figure 1:**
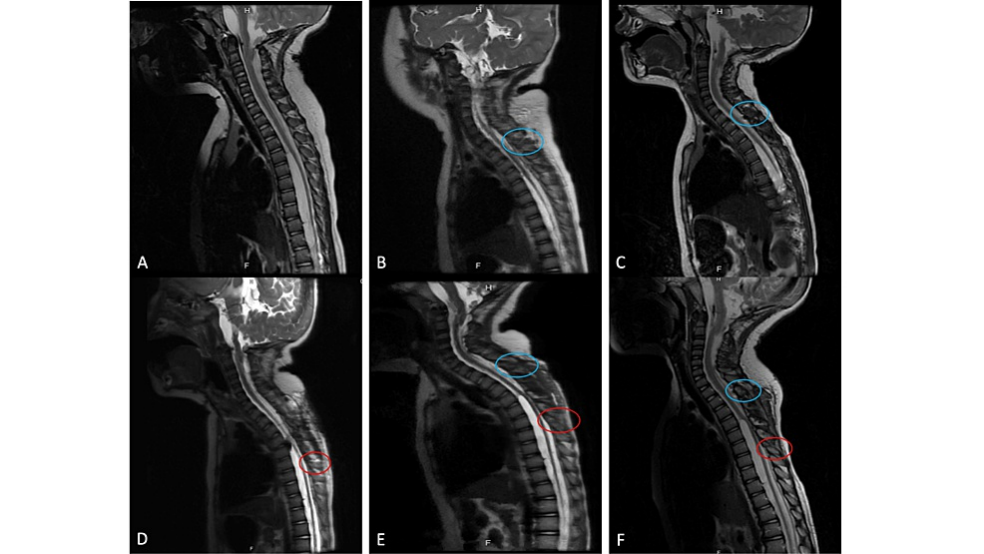
Case #1 MRI A-C: Preoperative MRI. Sagittal, T2-weighted MRI of the cervicothoracic spine demonstrating the ventral spinal arachnoid cyst. The cyst is large prior to his first cyst fenestration (A), surgically decompressed (B), and found to be re-expanding 7 months after his fenestration with increased dorsal displacement and deformation of the spinal cord (C). D-F: Post-operative MRI. Sagittal, T2-weighted MRI of the cervicothoracic spine demonstrating immediate post-operative changes (D) and healing of the spinal column at 1 and 3 months post-operatively. The colored circles indicate spinous processes that were split during his prior (blue) and presently described (red) surgeries. Transverse splitting of the spinous process can be seen on MRI as a linear increased T2 hyperintensity at the site of the split. Healing can be seen in subsequent post-operative scans as a resolution of this signal.

A 2-year-old male with a complex medical history is followed, more recently, for multiple ventral spinal arachnoid cysts (Fig. 1). He had previously undergone a thoracic two (T2) to T4 TSL for fenestration of the largest cyst and was progressing well with therapy. However, he experienced a functional decline 7 months after surgery, and imaging revealed an increase in the size of the thoracic cyst (Fig. 1C). The patient returned to the operating room for redo thoracic TSL, cyst fenestration, and placement of a syringo-subarachnoid shunt. The patient tolerated the procedure well, with an uncomplicated post-operative course, and was discharged on post-operative day 3. He was last seen in the clinic 3 months after surgery, progressing with therapy and healing appropriately (Fig. 1F).

Operative Technique

The patient was positioned prone in standard fashion. A skin incision was made through the inferior one-third of the patient’s prior incision, extending caudally by 2 cm. A sharp dissection was performed until we identified a fascial plane. Paramedian openings in the fascia were created, and subperiosteal dissection was used to expose the lamina.

**Figure 2:**
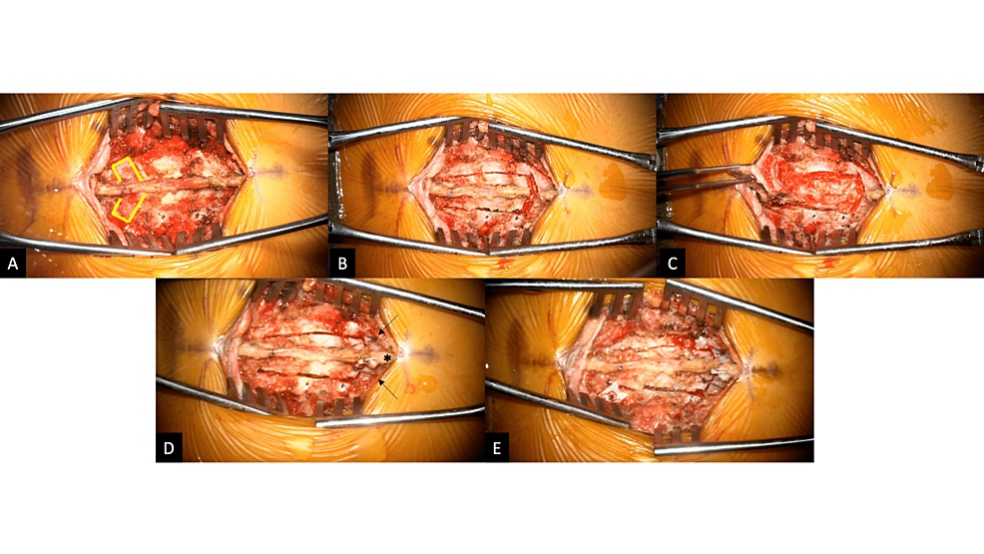
Intraoperative images from Case #1 The rostral aspect of the incision is to the left of the image. A: Exposure of the spinous processes and lamina of the thoracic spine. Resorbable plates and screws are seen at the bilateral lamina of T4 (yellow outline). B: The UBS was used to create laminectomies at the two rostral levels. Bilateral hemilaminotomies and split spinous process osteotomy were carried out at the most caudal level. The PTB remains intact rostrally. C: Hinging on the intact rostral PTB, the posterior elements are retracted rostrally exposing the underlying thecal sac and contents of the spinal canal. D: Once the intradural portion is completed, split lamina are reconstructed with purple-dyed 2-0 Vicryl sutures (black arrows), encircling the entire lamina, and a 2-0 FiberWire is used to re-approximate the split spinous process and supraspinous ligament (asterisk). E: Resorbable plates and screws are added at the laminectomy sites for reconstruction.

Upon exposure, we identified portions of resorbable plating that had previously been used to secure the lamina at T4 (Fig. 2A). Previous sites of laminectomy were well healed. The ultrasonic bone scalpel (UBS) was used to transversely split the lamina at the caudal most level of our exposure. The UBS was then used to create laminectomies at the two levels above this (Fig. 2B). The posterior elements were left attached rostrally and elevated to expose the underlying dura (Fig. 2C). The intradural portion of the procedure was accomplished without complication. The dura was closed in the usual fashion, and the posterior elements returned to their original position. The split lamina was re-approximated bilaterally, in a sublaminar fashion, using 2-0 Vicryl sutures (Ethicon, Inc., New Jersey, United States). The supraspinous ligament was re-approximated with FiberWire (Arthrex, Florida, USA) (Fig. 2D). Finally, the remaining lamina was reconstructed using resorbable plates and screws (Fig. 2E). The wound was closed in the usual fashion.

CASE #2

Clinical Presentation

**Figure 3:**
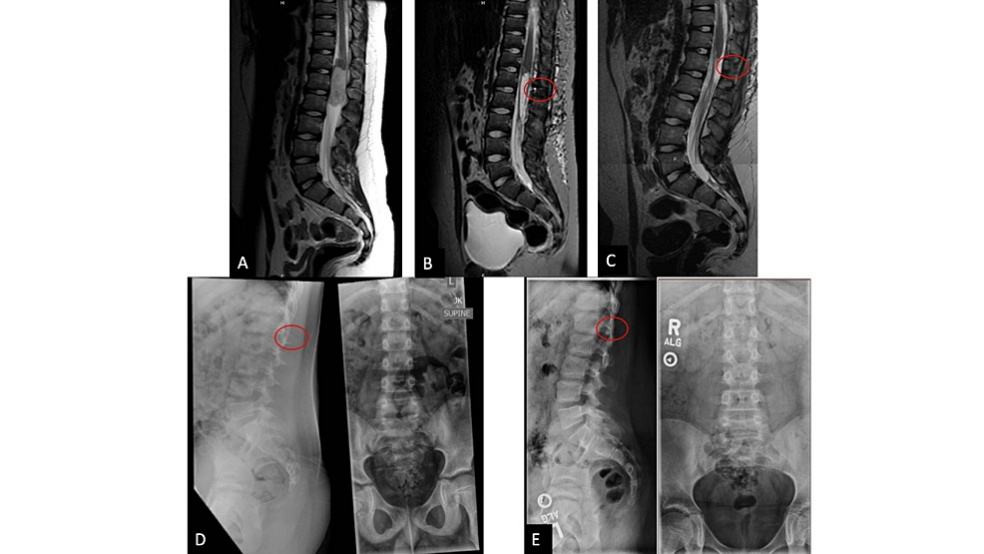
Case #2 pre- and post-operative imaging A-C: Sagittal, T2-weighted MRI of the thoracolumbar spine demonstrating the patient’s intradural extramedullary mass prior to resection (A). Subsequent images demonstrate the level of the transversely split spinous process and lamina (red circle), on post-operative day 1 (B) and 5 months post-operatively (C). D-E: Lateral and anteroposterior, upright, plain radiographs of the patient’s lumbar spine both pre-operatively (D) and 2.5 years post-operatively (E) demonstrate stable overall alignment. The level of the split posterior elements is identified by the red circle. Transverse splitting of the spinous process can be seen on MRI as a linear increased T2 hyperintensity at the site of the split. Healing can be seen in subsequent post-operative scans as the resolution of this signal.

A 9-year-old male with chronic right hip pain was found to have an intradural mass spanning T12 to lumbar two (L2) (Fig. 3). The patient underwent TSL for resection of the mass. The patient tolerated the procedure well, with an uncomplicated post-operative course, and was discharged on post-operative day 3. He has been followed routinely in the clinic for 2.5 years since surgery without clinical or radiographic evidence of deformity or instability (Fig. 3D-E).

Operative Technique

The patient was positioned prone in the usual fashion. The location of the skin incision and extent of exposure was verified using fluoroscopy. Sharp dissection was used to open the skin and reach the fascial layer. Paramedian openings in the fascia were created and subperiosteal dissection was used to expose the lamina.

**Figure 4:**
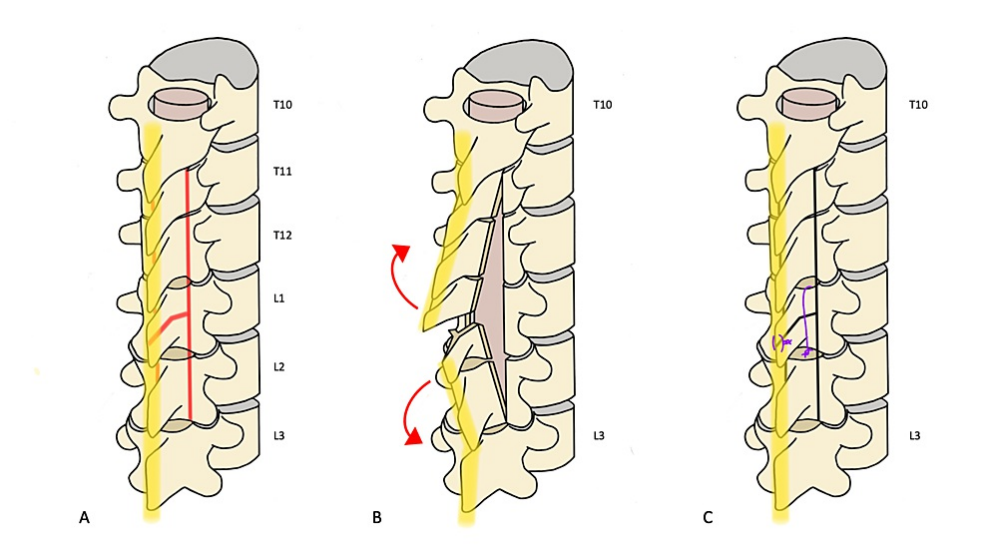
TSL technique as applied in Case #2 The yellow band represents the PTB in all panels. A: The spine is exposed from T10 to L3. The red lines represent osteotomies to be made using a UBS, with the transversely split lamina at L1. B: The laminectomies at T10 to L2 has been completed. Red arrows indicate the direction of boney retraction to access the spinal canal. C: The posterior elements have been reconstructed. Purple lines represent the sutures used to re-approximate the split lamina and spinous process. Black bold lines represent the sites of osteotomies. Reconstruction is completed with resorbable plating at the sites of laminectomy (not pictured here).

The UBS was used to transversely split the lamina and spinous process at L1. Laminectomies were completed at T11, T12, and L2. The posterior elements were elevated and retracted such that they separated at L1 while remaining attached at the rostral and caudal ends (Fig. 4). The intradural portion of the procedure was accomplished without complication. The dura was closed in the usual fashion, and the posterior elements returned to their original position. The split lamina was re-approximated in the same fashion as described in Case #1. The standard wound closure technique was followed.

## Discussion

Laminectomy and laminoplasty are the most common techniques for accessing the spinal canal through a posterior approach. A potential complication of multilevel laminectomies is the development of kyphotic deformity. Pediatric patients are at higher risk for developing post-laminectomy spinal deformities due to increased viscoelasticity of ligaments and more horizontally oriented facet joints in the context of a continually growing, biomechanically altered spine [2]. There is conflicting evidence regarding the benefits of laminoplasty versus laminectomy in preventing post-operative deformities [3-5]. Despite this, it is theorized that with laminoplasty, the restoration of the PTB, and bony refusion, spinal integrity is re-established, thus decreasing the risk of the development of spinal deformity.

We believe that our novel technique, as illustrated here, more effectively achieves this goal of restoring the patient’s native anatomy. The most similar previously described technique involves hemilaminectomies at both the rostral and caudal-most levels for the complete removal of intervening bone [6]. Our technique takes one step further in preserving the PTB by preserving the ligamentum flavum, interspinous and supraspinous ligaments at all, but one end, of the exposure. At the site of the split laminotomy, only the supraspinous ligament is disrupted. In cases, such as patient 2, the level of the transversely split lamina may be planned in the middle of the bony exposure to facilitate the further extension of the exposure rostrally or caudally (Fig. 4). Another point to take note of our operative technique is the paramedian fascial incision used on either side of the PTB. This maximizes the amount of tissue that is left preserved while still enabling subperiosteal dissection down to bone and water-tight re-approximation of the fascial layer. We also utilized resorbable plating systems for the youngest age group which offers several benefits: rigid fixation that sutures cannot, alternative options when there is limited bony exposure to place holes to accommodate suture, and a reduction in operative time. Finally, we advocate for use of a UBS to minimize bone loss and blood loss while minimizing the risks of dural tears [6-9].

## Conclusions

TSL is an effective alternative to conventional laminoplasty for accessing the intraspinal space. Potential limitations of this technique may arise depending on the patient's age. The size of the adjacent spinous processes may inhibit hinging of lamina segments in older patients, while infant lamina may be too narrow to split. Therefore, as with all surgical techniques, patient selection plays a role. Overall, this technique minimally disrupts the soft tissue anatomy and relies primarily on osteogenesis for more optimal healing and preservation of normal anatomy.

## References

[1] Yasuoka S, Peterson HA, MacCarty CS (1982). Incidence of spinal column deformity after multilevel laminectomy in children and adults. J Neurosurg.

[2] Krishnan A, Samal P, Mayi S, Degulmadi S, Rai RR, Dave B (2021). Thoracic spine stenosis: does ultrasonic osteotome improve outcome in comparison to conventional technique?. Malays Orthop J.

[3] Lin Q, Lin T, Wang Z, Chen G, Liu W (2022). Safety and effectiveness of modified expansive open-door laminoplasty using a ultrasonic bone scalpel compared with a high-speed drill. Clin Spine Surg.

[4] Frank CB (2004). Ligament structure, physiology and function. J Musculoskelet Neuronal Interact.

[5] McGirt MJ, Garcés-Ambrossi GL, Parker SL (2010). Short-term progressive spinal deformity following laminoplasty versus laminectomy for resection of intradural spinal tumors: analysis of 238 patients. Neurosurgery.

[6] Önen MR, Naderi S (2021). Bone-to-bone ligament preserving laminoplasty technique for reconstruction of laminae. J Craniovertebr Junction Spine.

[7] Wahlquist S, Nelson S, Glivar P (2019). Effect of the ultrasonic bone scalpel on blood loss during pediatric spinal deformity correction surgery. Spine Deform.

[8] McGirt MJ, Chaichana KL, Atiba A, Bydon A, Witham TF, Yao KC, Jallo GI (2008). Incidence of spinal deformity after resection of intramedullary spinal cord tumors in children who underwent laminectomy compared with laminoplasty. J Neurosurg Pediatr.

[9] Yasuoka S, Peterson HA, Laws ER Jr, MacCarty CS (1981). Pathogenesis and prophylaxis of postlaminectomy deformity of the spine after multiple level laminectomy: difference between children and adults. Neurosurgery.

